# Under-Recognized Pathogens in Peritoneal Dialysis Associated-Peritonitis: The Importance of Early Detection

**DOI:** 10.7759/cureus.35445

**Published:** 2023-02-25

**Authors:** Patrícia Domingues, Teresa Furtado, Patrícia Valério, José Matias, Liliana Cunha

**Affiliations:** 1 Nephrology, Setubal Hospital Center, Setubal, PRT; 2 Nephrology, Centro Hospitalar De Setubal, Setubal, PRT

**Keywords:** gram negative infection, end-stage kidney disease (eskd), prevotella, peritonitis, peritoneal dialysis

## Abstract

Peritoneal dialysis-associated-peritonitis remains a major concern, increasing patient morbidity and mortality. Empirical antibiotics should be quickly started to allow a rapid resolution of symptoms and preservation of the peritoneal membrane.

We report a case of peritoneal dialysis-associated-peritonitis due to *Prevotella* *salivae* and *Corynebacterium jeikeium*, in a 51-year-old male. Suspected peritonitis led to an immediate prescription of vancomycin and ceftazidime, with no clinical improvement. *Prevotella* is difficult to identify in culture since it’s a gram-negative anaerobic bacterium, so metronidazole administration was delayed over days.

New diagnostic techniques have been explored for the early diagnosis of peritonitis, including polymerase chain reaction (PCR) for bacterial DNA fragments. A multiplex PCR panel that includes *Prevotella*, already available for other applications, could be an advantage in cases like this.

## Introduction

Peritonitis remains a major complication of peritoneal dialysis (PD) [1]. Despite PD peritonitis rates decreasing over time, Europe still has an estimated rate of 0.303 episodes/patient-year in 2019 [2]. A rapid presumptive diagnosis is essential because a delay in treatment is related to high morbidity and mortality [1,2].^ ^The diagnosis can be suspected when [1]:

- clinical features consistent with peritonitis are present (abdominal pain and/or cloudy dialysis effluent);

- dialysis effluent white cell count is greater than 100/mL (or greater than 0.1 x 10^9^/L, after a dwell time of at least two hours), with more than 50 percent of polymorphonuclear cells (PMN)

- when there is a positive dialysis effluent culture.

As recommended by the International Society of PD (ISPD) peritonitis guideline, two or more criteria are required for PD-associated-peritonitis diagnosis [1].

An appropriate method for PD effluent culture is the most important step to identify the causative organism. Nevertheless, PD effluent cultures can be negative for several reasons, namely recent antibiotic exposure, suboptimal specimen collection, fungal and mycobacterial peritonitis, or misclassification [1].

Empirical antibiotics should be started to allow a faster resolution of inflammation and abdominal pain, and also to preserve the peritoneal membrane [2].^ ^Apart from the direct impact on patient survival, it remains the primary reason for changing dialysis modality from PD to hemodialysis [3,4].

## Case presentation

We report the case of a 51-year-old male with a history of long-term hypertension, immunity to hepatitis B virus due to exposure, chronic kidney disease due to anti-glomerular basement membrane disease on intermittent PD program for one year, and secondary hyperparathyroidism. PD was complicated by past infection complications: three episodes of exit-site infections, with detection of meticillin-sensitive *Staphylococcus aureus* (MSSA), which lead to external cuff catheter extrusion; one episode of combined exit-site, tunnel infection, and peritonitis due to MSSA; peritonitis due to *Streptococcus mitis*; and exit-site infection due to *Corynebacterium amycolatum*. Infections are described in Table 1.

**Table 1:** History of infections over time. MSSA - Methicillin Sensitive *Staphylococcus aureus*; IV – intravenous.

Time	Type of infection	Isolated B acteria	Antibiotic	Time of therapy	Administration
September 2021	Exit-site infection	MSSA	Flucloxacilin	2 weeks	Oral
October 2021	Exit-site infection	MSSA	Flucloxacilin	3 weeks	Oral
November 2021	Exit-site infection	MSSA	Flucloxacilin + Vancomicin	6 weeks	IV
February 2022	Peritonitis + Tunelitis + Exit-site infection	MSSA	Vancomicin à Flucloxacilin	3 weeks	IV à Oral
May 2022	Peritonitis + Exit-site infection	Streptococcus mitis	Vancomicin + Ceftazidime à Vancomicin	3 weeks	IV
June 2022	Exit-site infection	Corynebacterium amycolatum	Gentamicin + Amoxicilin + Clavulanate	2 weeks	Topic + Oral

One month after his last infection, treated with a two weeks regimen of oral amoxicillin/ clavulanate and topical gentamicin (according to an antibiotic sensitivity test), he presented with abdominal pain and cloudy effluent.

An empiric intraperitoneal antibiotic was started (vancomycin and ceftazidime) after the effluent leukocytes count result (810/mL with 85% of PMN cells). On the next day, the peritoneal catheter was dysfunctional because it was misplaced (the position was confirmed by abdominal x-ray), with no improvement despite the laxatives prescription. Therefore, the patient started hemodialysis (HD) by central catheter, with antibiotic administration after sessions.

Ten days after initial antibiotic treatment, he was admitted to the hospital with intense abdominal pain located at the exit site of the peritoneal catheter. Another intra-abdominal disease was excluded. The catheter was removed since he had a previous episode of catheter-related infection, a history of recurrent infection, intense pain, and increased levels of inflammatory markers (11×10^9^/L leukocytes, C-reactive protein of 42,7 mg/dL).

Dialysis effluent culture was delayed since the first culture was polymicrobial and a repeated culture was required. *Corynebacterium jeikeium* and *Prevotella salivae* bacteria were identified, and no sensitivity antibiotic test was provided. PD catheter culture did not isolate any microorganisms.

Worsening of the patient’s general condition without laboratory improvement led to metronidazole administration, eleven days after initial therapy with vancomycin and ceftazidime. The patient was able to recover after completing seven days of metronidazole and seventeen days of vancomycin and ceftazidime.

## Discussion

Corynebacterium species have been considered harmless, but nowadays they are recognized as responsible for gram-positive infections, including, PD peritonitis [1,5].^ ^Although typically susceptible to vancomycin, *Corynebacterium spp.* are considered at moderate risk for requiring catheter removal [1,5]. Improved recognition of its pathogenicity allows for increased identification and treatment.

*Prevotella salivae* is an anaerobic gram-negative bacillus, commonly associated with oral and gastrointestinal infections, chronic osteomyelitis, bite-related infections, or rheumatoid infections. This bacterium can produce beta-lactamase, making Ceftazidime (cephalosporin antibiotic) a poor option, for this reason, metronidazole was started, the recommended antibiotic for empiric uses in these cases [6].

Antibiotic therapy according to the susceptibility of isolated microorganisms is the preferable approach. However, Prevotella, as an anaerobic gram-negative, represents a challenge for identifying, requiring the expertise of the laboratory [7,8].^ ^Methods to test the susceptibility of anaerobes are well standardized, but they are difficult and most of the time they are not cost-effective since most anaerobes have predictable susceptibility profiles [8]. Some studies propose that the development of a multiplex polymerase chain reaction (PCR) panel could have an important impact on detecting anaerobic bacterial [7,9].^ ^This method enables faster detection of anaerobes, overcoming difficulties related to anaerobic detection based on culture [7,9].^ ^Besides that, multiplex PCR could also be useful in antibiotic-resistant Prevotella, identifying genes involved in decreased susceptibility of Prevotella to metronidazole, however, not all resistance mechanisms are genetically dependent [10].

## Conclusions

Identifying and recognizing the potential pathogenicity of Prevotella salivae during the interpretation of culture results is the first step for a successful treatment. Nephrologists should be aware of this possibility. Future use of multiplex PCR technique could be the answer for an early diagnosis and treatment anticipation with metronidazole.
